# Donafenib intolerance in hepatocellular carcinoma: severe hand–foot skin reaction and successful switch to lenvatinib – a case report and literature review

**DOI:** 10.3389/fonc.2025.1728098

**Published:** 2025-12-18

**Authors:** Ying Chen, Linglin Fu, Ping Song, Yinuo Tan, Yuqi Jin

**Affiliations:** 1 Department of Nursing, The Second Affiliated Hospital, Zhejiang University School of Medicine, Hangzhou, Zhejiang, China; 2 School of Renji Medical Sciences, Wenzhou Medical University, Wenzhou, China; 3 Department of Medical Oncology, Key Laboratory of Cancer Prevention and Intervention, Ministry of Education, The Second Affiliated Hospital, Zhejiang University School of Medicine, Hangzhou, Zhejiang, China; 4 Zhejiang Provincial Clinical Research Center for Cancer, Hangzhou, China; 5 Cancer Center of Zhejiang University, Hangzhou, China; 6 Center for Medical Research and Innovation in Digestive System Tumors, Ministry of Education, Hangzhou, China

**Keywords:** donafenib, hepatocellular carcinoma, hand–foot skin reaction, acral toxicity, lenvatinib, tyrosine kinase inhibitor, multikinase inhibitor, case report

## Abstract

**Background:**

Donafenib is an approved multikinase inhibitor for hepatocellular carcinoma (HCC). However, cutaneous toxicity—particularly hand–foot skin reaction (HFSR)—may necessitate treatment interruption and compromise therapeutic continuity.

**Case presentation:**

A 58-year-old man with HCC on a cirrhotic background developed abrupt onset of intensely painful plantar erythema with overlying desquamation 10–11 days after initiating donafenib. The lesions rapidly progressed, leading to impaired ambulation and were consistent with CTCAE grade 3 HFSR.

**Management and outcome:**

Donafenib was immediately discontinued, and the patient received short-term symptomatic management, resulting in prompt improvement of the acral lesions. He was subsequently transitioned to lenvatinib, which was well tolerated without recurrence of high-grade skin toxicity. The patient maintained clinical stability and was able to continue systemic anticancer therapy.

**Conclusion:**

This case highlights the importance of early detection and accurate grading of HFSR, timely treatment interruption, and mechanism-informed switching to an alternative tyrosine kinase inhibitor such as lenvatinib. It also underscores key differences in toxicity profiles between donafenib—associated with VEGFR/RAF-related cutaneous injury—and lenvatinib, which is more commonly linked to hypertension, diarrhea, and appetite or weight changes.

## Introduction

Hepatocellular carcinoma (HCC) remains a leading cause of cancer-related mortality worldwide, and many patients present at stages unsuitable for curative surgery or ablation (1, 2). For these patients, systemic therapy is central to care. Multikinase inhibitors (MKIs) that target vascular endothelial growth factor receptors (VEGFRs), fibroblast growth factor receptors (FGFRs), and RAF kinases have become established treatment options and are widely used either as monotherapy or in combination with locoregional approaches (3, 4). Donafenib, a deuterated analog of sorafenib, is one such agent and is increasingly adopted in routine practice (5).

Dermatologic adverse events—particularly hand–foot skin reaction (HFSR)—are among the most frequent and function-limiting toxicities of MKIs (6–9). These reactions typically emerge early, involve pressure-bearing acral skin, and may result in dose reduction, treatment interruption, or permanent discontinuation. Practical guidance emphasizes prompt assessment, friction avoidance, liberal use of emollients and keratolytics, and appropriate topical or short courses of systemic corticosteroids. When symptoms are severe or recur despite optimal supportive care, switching to an alternative regimen with a different adverse event profile is considered a reasonable strategy (10, 11).

Donafenib inhibits VEGFR1–3, PDGFR, and RAF kinases, exerting both antiangiogenic and antiproliferative effects. Deuterium substitution at key metabolic sites slows oxidative metabolism via CYP3A4 and glucuronidation through UGT1A9, prolonging drug stability and potentially improving tolerability relative to sorafenib (12, 13). Lenvatinib, another oral MKI, targets VEGFR1–3, FGFR1–4, PDGFRα, RET, and KIT while sparing RAF signaling. This pharmacologic distinction translates into differing toxicity profiles: hypertension, proteinuria, and gastrointestinal effects are more characteristic of lenvatinib, whereas RAF inhibition–related hyperkeratotic HFSR is more strongly associated with sorafenib-like agents (14).

Here, we describe an early, disabling acral reaction occurring shortly after donafenib initiation in a patient with HCC and cirrhosis, outline the clinical decision-making that led to drug discontinuation and switching to lenvatinib, and summarize key considerations to help clinicians balance toxicity management with the need to maintain effective anticancer therapy.

## Case presentation

A 58-year-old man was admitted on 11 February 2025 after a routine health examination performed five days earlier identified a hepatic mass. He was alert and hemodynamically stable. His medical history was notable for liver cirrhosis, splenomegaly, multiple hepatic cysts, and a left renal cyst, with no personal or family history of malignancy. Contrast-enhanced upper-abdominal magnetic resonance imaging (MRI) on 6 February 2025 revealed two enhancing nodules in the right hepatic lobe, the largest measuring 24 × 27 mm, on a cirrhotic background. A repeat MRI on 8 February 2025 again favored hepatocellular carcinoma involving segments VII/VIII, with an additional arterial-phase enhancing lesion in segment V.

After multidisciplinary evaluation, the patient underwent transcatheter arterial chemoembolization (TACE) on 14 February 2025 without complications. He was discharged the following day and started on donafenib 200 mg twice daily on 15 February 2025. Approximately ten days after discharge, on 25 February 2025, he developed painful erythematous lesions on the plantar surfaces. By the next day, his pain limited ambulation, prompting admission to The First People’s Hospital of Wenling, where he remained from 26 February to 3 March. Treatment there included single intravenous doses of dexamethasone and chlorpheniramine, calcium gluconate infused over roughly twenty minutes, and short courses of reduced glutathione and filgrastim administered empirically for a possible inflammatory or marrow-suppression component. Despite these measures, sheet-like plantar desquamation progressed by 28 February, coinciding with day 13 of donafenib exposure.

After returning to our center, dermatologic examination revealed well-demarcated, callus-like hyperkeratosis with extensive desquamation and fissuring across pressure-bearing palmoplantar sites, without any mucosal involvement. Based on the morphology, anatomic distribution, and pain severe enough to impair walking, the presentation was consistent with multikinase-inhibitor–associated hand–foot skin reaction, classified as Common Terminology Criteria for Adverse Events (CTCAE) v5.0 grade 3. Donafenib was discontinued on 3 March 2025. Supportive management included pressure off-loading using insoles and silicone pads, nightly application of urea-based keratolytics, and brief pulses of high-potency topical corticosteroids such as clobetasol 0.05% during symptomatic flares.

**Figure 1:**
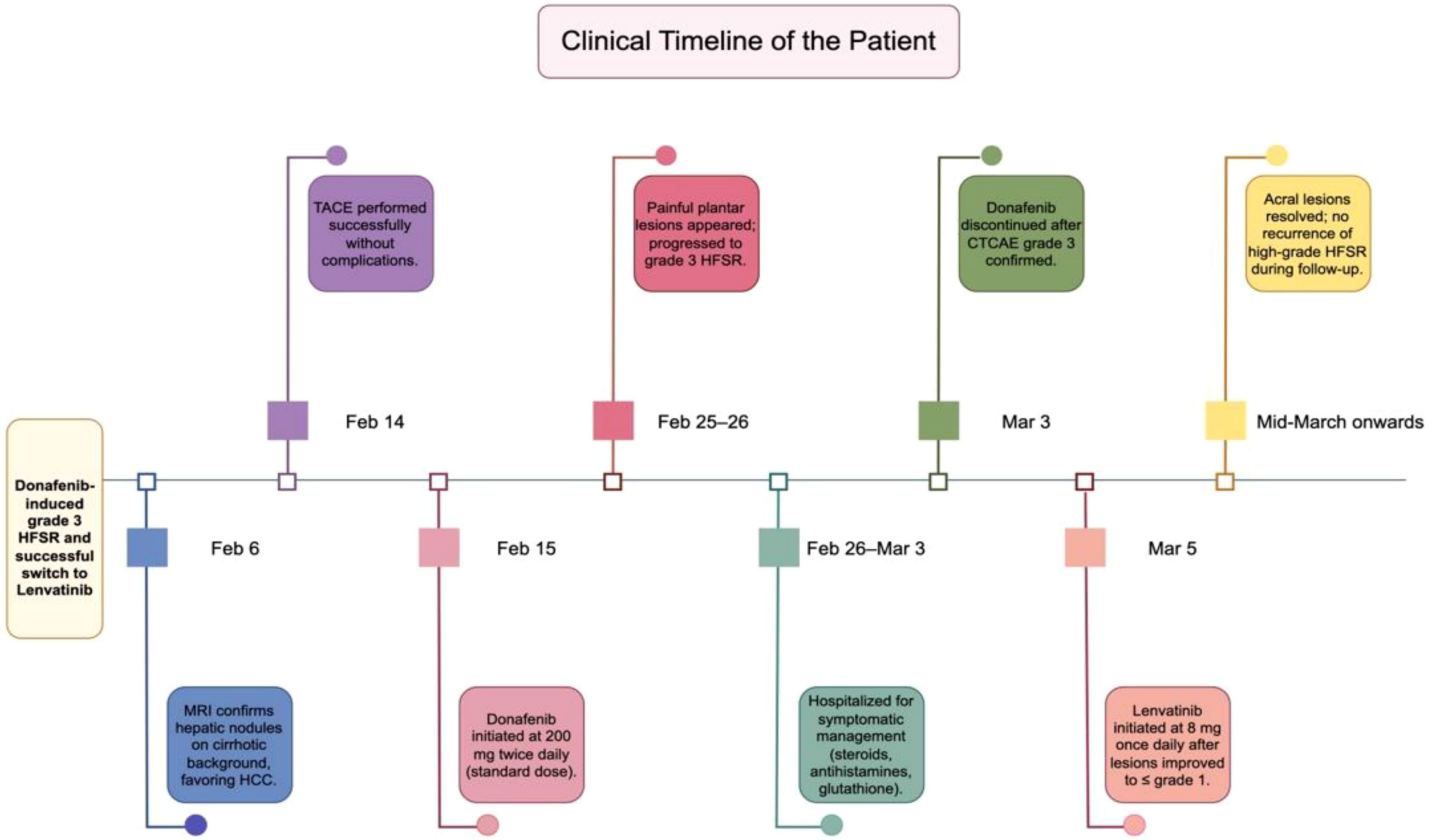
Clinical timeline of the patient.

**Figure 2:**
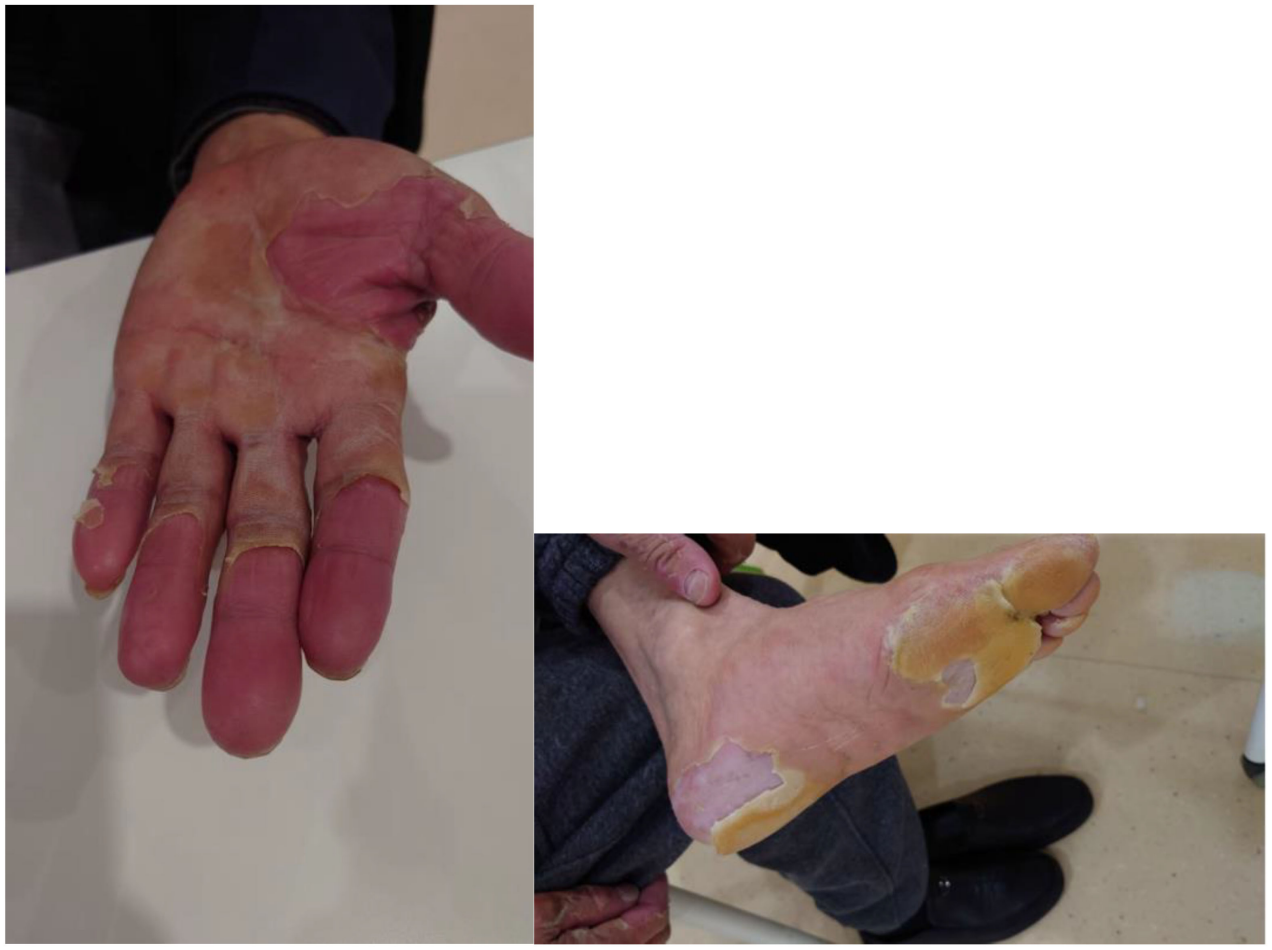
Day 11 after donafenib initiation.

**Figure 3:**
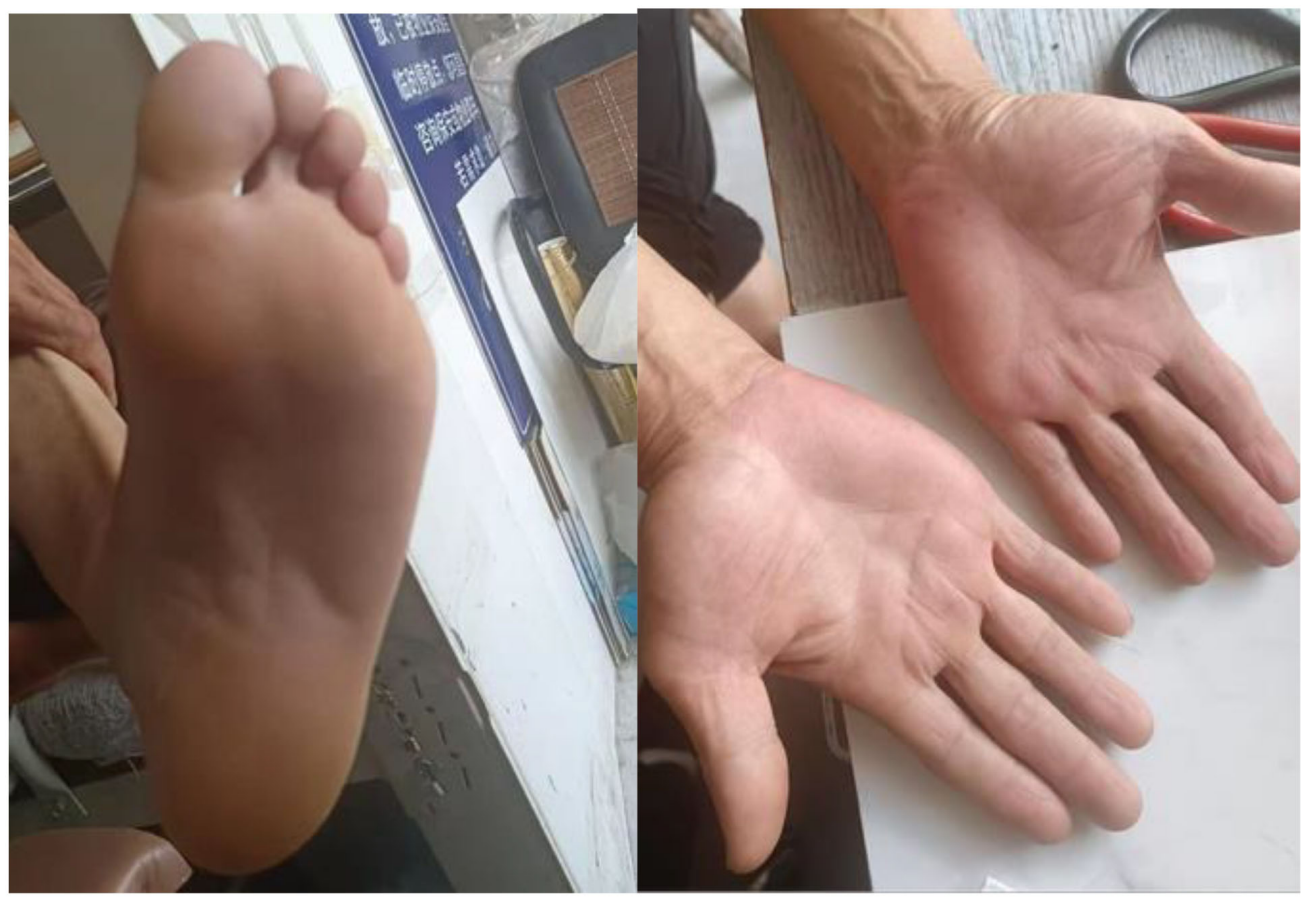
Post-switch follow-up after lenvatinib.

After the lesions improved to grade 1 or lower in early March, systemic therapy was transitioned to lenvatinib at a dose of 8 mg once daily, initiated on 5 March 2025 according to the patient’s body weight (65 kg). Over the ensuing weeks, pain and hyperkeratotic plaques progressively resolved, and no further dermatologic complications occurred. During lenvatinib therapy, monitoring focused on blood pressure and urinary protein levels in accordance with its established toxicity profile. A detailed clinical timeline is presented in **Figure 1** (made by figdraw), with serial photographs documenting clinical improvement shown in **Figures 2**, **3**.

## Literature review

Using the search term “Donafenib” in combination with “hepatocellular carcinoma,” we reviewed clinical trials, real-world series, and case reports; key efficacy and safety data are summarized in **Supplementary Table 1**, with practical contrasts versus Lenvatinib presented in **Table 1**. Donafenib is a deuterated analog of sorafenib that inhibits RAF kinases together with VEGFR, PDGFR, and KIT. Deuteration slows oxidative metabolism and may flatten exposure–time profiles, but in cirrhosis the combination of reduced clearance and altered protein binding can still yield higher effective exposure at standard doses (15). This pharmacology underlies both its antitumor activity and the inter-individual variability in tolerability observed across studies.

**Table 1:** Comparative summary of donafenib and lenvatinib: efficacy, safety, and cost-effectiveness findings from published analyses.

Study (Journal/Year)	Comparison focus	Key findings (Concise)	OS/PFS	Cost-Effectiveness	Adverse events/safety	Citation
Therap Adv Gastroenterol, 2022 (Sun et al.)	NMA + cost-effectiveness (China/USA)	At baseline WTP thresholds, Lenvatinib is more cost-effective; in low-income scenarios, Donafenib is most cost-effective.	ICI + anti-VEGF combos outperform TKI monotherapy in OS/PFS; Lenvatinib ranks high for PFS.	Baseline: LEN favored; Low-income scenario: DON favored.	No definitive conclusion that LEN has higher AEs than DON.	(23)
Expert Rev Pharmacoecon Outcomes Res, 2022 (Meng et al.)	DON vs LEN cost-effectiveness	DON more cost-effective than LEN; model: ΔQALY≈+0.139, Δcost≈+$1,500, ICER≈$10,790/QALY; CE probability≈84.9% at China threshold.	Based on indirect inputs (ZGDH3, REFLECT); no new direct OS/PFS comparison.	DON favored (China payer perspective).	Economic focus; no strong AE conclusion.	(24)
Front Public Health, 2022 (Zhao et al.)	Economic evaluation of five first-line regimens	Atezolizumab+Bevacizumab best on effectiveness; DON most economical at then-current prices and thresholds.	Combinations lead OS/PFS overall.	DON most economical (China threshold).	Not a primary endpoint.	(25)
Advances in Therapy, 2022 (Guan et al.)	DON vs LEN vs SOR cost-effectiveness	DON achieved highest QALYs and lowest cost; more cost-effective than LEN and SOR in China.	Not a primary endpoint (NMA inputs).	DON favored over LEN and SOR (China).	Not a primary endpoint.	(26)
Eur J Cancer, 2022 (Fulgenzi et al.)	NMA of landmark phase III first-line trials	ICI + anti-VEGF combinations (e.g., atezolizumab+bevacizumab) superior in OS/PFS to TKI monotherapy.	Combinations overall best; LEN among top for PFS.	Not assessed.	Safety profiled; no conclusion that LEN > DON for AEs.	(27)
Front Oncol, 2021 (Liu et al., Dec 24)	NMA of first-line systemic therapies	Combinations overall best; LEN ranks among top for PFS; DON superior to SOR for OS.	As at left.	Not assessed.	No definitive conclusion that LEN has higher AEs than DON.	(28)
World J Gastroenterol, 2021 (Han et al.)	NMA of first-line RCTs	Consistent with later NMAs: combinations superior to TKI monotherapy; LEN ranks high for PFS.	Combinations superior to TKI monotherapy.	Not assessed.	No clear LEN > DON AE conclusion.	(29)

Comparative data are derived from indirect analyses; no head-to-head clinical trials between Donafenib and Lenvatinib are currently available.

DON, donafenib; LEN, lenvatinib; SOR, sorafenib; ICI, immune checkpoint inhibitor; VEGF, vascular endothelial growth factor; OS, overall survival; PFS, progression-free survival; QALY, quality-adjusted life year; ICER, incremental cost-effectiveness ratio; WTP, willingness-to-pay; AE, adverse event.

Across randomized and observational cohorts, Donafenib has shown consistent disease-control and survival benefits compared with sorafenib in Chinese populations. Because head-to-head trials with Lenvatinib are lacking, practice tends to pivot instead on baseline hepatic reserve and the anticipated toxicity profile. Donafenib more commonly produces acral, hyperkeratotic HFSR, whereas Lenvatinib is more often associated with hypertension, proteinuria, diarrhea, and appetite or weight changes (5). Combination regimens and prior locoregional interventions may further amplify hepatotoxicity and hematologic abnormalities, particularly in patients with cirrhosis, underscoring the need for dose adjustment and close biochemical monitoring (16, 17). Our case is consistent with this clinical pattern: painful, well-demarcated plantar lesions developed within two weeks of Donafenib initiation and improved after treatment interruption, compatible with exposure-related HFSR in pressure-bearing skin. Switching to Lenvatinib then allowed continued anti-angiogenic therapy with improved cutaneous tolerability, reflecting the distinct molecular targets of these two TKIs.

Mechanistically, the contrast between Donafenib and Lenvatinib provides a plausible explanation for their divergent dermatologic profiles. Donafenib inhibits RAF kinases in addition to VEGFR-2/3, producing potent blockade of the VEGF–MAPK axis in dermal microvasculature and keratinocytes (18). In friction-rich, eccrine-dense acral skin, this dual signal interruption may promote microvascular injury and keratinocyte stress, leading to ischemic inflammation and the hyperkeratosis characteristic of HFSR. Cirrhosis-related hypoalbuminemia and impaired clearance may further increase effective exposure and risk (19, 20). By contrast, Lenvatinib targets VEGFR-1/2/3 and FGFR-1–4, with additional activity against PDGFR-α, RET, and KIT, but does not directly inhibit RAF. As a result, MAPK-driven keratinocyte stress is attenuated and endothelial injury tends to remain subclinical, shifting the toxicity profile toward hypertension, proteinuria, and gastrointestinal events rather than prominent acral hyperkeratosis. HFSR can still occur with Lenvatinib but appears less frequent and generally less severe than with RAF-inhibiting multikinase inhibitors (21, 22).

In essence, Donafenib’s VEGFR–RAF co-blockade creates local microvascular fragility under mechanical load, whereas Lenvatinib’s RAF-sparing profile produces a more systemic, non-cutaneous pattern of adverse events, thereby supporting a rational switch strategy in patients who develop severe acral toxicity.

## Discussion

This case illustrates an early-onset, function-limiting acral toxicity during donafenib therapy for HCC, with symptom onset around day 10 and rapid progression to sheet-like plantar desquamation. The close temporal relationship to drug initiation, the characteristic distribution on pressure-bearing skin without mucosal involvement, and resolution after drug withdrawal followed by a switch to lenvatinib support a probable causal association with donafenib-induced HFSR (CTCAE v5.0 grade 3). Although a drug-induced allergic eruption cannot be completely excluded in the absence of standardized dermatologic photography, the morphology—well-demarcated hyperkeratosis and fissuring on the palmoplantar surfaces—together with pain-limited ambulation is more typical of HFSR than of morbilliform exanthema or severe hypersensitivity syndromes (30, 31).

Differential diagnoses included post-embolization dermatitis after TACE and severe cutaneous adverse reactions (SCARs) such as drug reaction with eosinophilia and systemic symptoms (DRESS) and Stevens–Johnson syndrome/toxic epidermal necrolysis (SJS/TEN). The focal, callus-accentuated plantar distribution and the absence of fever, facial edema, eosinophilia, or mucosal erosions argued against these entities (32–34). Fluctuations in liver enzyme levels around the time of surgery were temporally distinct from the cutaneous course and therefore unlikely to account for the skin findings (35).

Mechanistically, multikinase inhibitor–induced HFSR likely reflects on-target inhibition of the VEGF–MAPK axis in eccrine-rich, high-friction acral skin. VEGFR blockade compromises the dermal microvasculature, while RAF/MAPK suppression heightens keratinocyte stress, so mechanical load tips the balance toward localized ischemic–inflammatory injury and hyperkeratosis—hallmarks of HFSR seen with sorafenib-class agents and relevant to donafenib (36). In patients with cirrhosis, reduced drug clearance and hypoalbuminemia can increase effective exposure at a given nominal dose, plausibly lowering the threshold for severe toxicity (37, 38). Guided by this biology, we used a stepwise bundle—pressure off-loading, emollients with keratolytics, short pulses of high-potency topical corticosteroids, and temporary interruption at grade 3—followed by a switch to lenvatinib once lesions had improved to grade ≤1, in order to preserve anticancer intent (32, 38).

Lenvatinib sustains anti-angiogenic pressure via VEGFR1–3 and FGFR1–4 but does not inhibit RAF, a profile that in trials and reviews aligns with a toxicity pattern dominated by hypertension, proteinuria, and gastrointestinal effects, with less prominent HFSR than RAF-inhibiting MKIs (39–42); this difference explains the improved cutaneous tolerability we observed. Pharmacologically, donafenib is a deuterated analog of sorafenib that inhibits VEGFR1–3, PDGFR, and RAF kinases, producing potent anti-angiogenic and antiproliferative effects (12, 13). However, concurrent RAF inhibition has been associated with a higher incidence of dermatologic toxicities such as HFSR. In contrast, lenvatinib targets VEGFR1–3, FGFR1–4, PDGFRα, RET, and KIT while sparing RAF signaling. This distinct kinase-inhibition profile allows it to maintain anti-angiogenic efficacy while reducing the likelihood of callus-type acral inflammation and keratinocyte stress associated with donafenib. These pharmacologic differences explain the improved tolerability observed after switching and provide a mechanistic justification for selecting lenvatinib as an alternative TKI in patients with donafenib intolerance. The core molecular targets, characteristic adverse event profiles, and practical management considerations for donafenib and lenvatinib are summarized in **Table 2**.

**Table 2:** Molecular targets, adverse-event profiles, and management considerations of donafenib and lenvatinib.

Item	Donafenib	Lenvatinib
Core targets	VEGFR1–3, PDGFR; RAF (sorafenib-like, deuterated analogue)	VEGFR1–3; FGFR1–4; PDGFRα; RET; KIT
Typical dermatology	HFSR more frequent: occurs on pressure/friction sites; well-demarcated, callus-like plaques; rash/erythema may also appear	HFSR can occur but is usually not dominant; pruritus/rash may be seen
Non-dermatologic adverse events (AEs)	Hypertension; fatigue; gastrointestinal events (diarrhea, appetite loss); laboratory abnormalities	Hypertension, diarrhea, appetite/weight change, proteinuria; fatigue
Practical notes	For refractory grade ≥3 HFSR: interrupt therapy and give supportive care; once lesions improve to ≤ grade 1, consider switching to Lenvatinib	Prioritize early management and follow-up of blood pressure and proteinuria; if hypertension/diarrhea dominate and remain difficult to control, dose-reduce or interrupt per guidelines

Comparative data are derived from indirect analyses; no head-to-head clinical trials between Donafenib and Lenvatinib are currently available.

Clinically, this case highlights several practical points. First, early recognition and accurate grading of acral pain and hyperkeratotic plaques during the first two weeks of therapy are crucial, as timely treatment interruption can prevent progression to disabling lesions. Second, a mechanism-based supportive approach—combining pressure off-loading, keratolytic agents, and short courses of potent topical corticosteroids—can effectively control symptoms and accelerate recovery. Third, when toxicity reaches grade 3 or markedly impairs function, switching to an alternative tyrosine kinase inhibitor (TKI) with a distinct toxicity profile (in this case, weight-based lenvatinib at 8 mg once daily) is a rational strategy that allows patients to continue systemic therapy without recurrence of high-grade cutaneous events.

The main limitations of this report include the lack of standardized lesion photography and dermatopathological confirmation, which preclude definitive phenotypic characterization. Moreover, because this is a single-patient observation, unmeasured confounders cannot be completely excluded. Nevertheless, the chronological sequence (drug initiation → symptom onset → treatment interruption → improvement → successful switch), objective clinical evaluations, and the absence of alternative diagnoses support a coherent causality narrative.

In summary, donafenib remains an important therapeutic option for HCC; however, clinicians should be aware that severe HFSR can develop early and significantly impair daily function. In this case, prompt recognition and grading, timely interruption, structured supportive management, and an individualized switch to lenvatinib enabled complete symptom resolution while maintaining anticancer treatment. Given the patient’s cirrhotic background, continued monitoring for lenvatinib-specific risks, such as QT-interval prolongation and hypertension, is recommended.

## Conclusion

Early recognition and grading of donafenib-induced hand–foot skin reaction (HFSR), followed by timely treatment interruption and an individualized switch to lenvatinib, enabled continuation of systemic therapy and complete symptom resolution in this cirrhotic HCC patient. This case underscores the importance of mechanism-based management and rational within-class switching for patients with multikinase inhibitor intolerance.

## Patient perspective

After experiencing severe pain and walking difficulty due to hand–foot skin reaction, the patient expressed relief and gratitude following prompt management and recovery. He reported satisfaction with the improvement of symptoms after switching to Lenvatinib and expressed confidence in continuing treatment under close medical supervision.

## Data Availability

The original contributions presented in the study are included in the article/**Supplementary Material**. Further inquiries can be directed to the corresponding authors.

## References

[1] Llovet JM Kelley RK Villanueva A Singal AG Pikarsky E Roayaie S . Hepatocellular carcinoma. Nat Rev Dis Primers. (2021) 7:6. doi: 10.1038/s41572-020-00240-3, PMID: 33479224 PMC13537433

[2] Sung H Ferlay J Siegel RL Laversanne M Soerjomataram I Jemal A . Global cancer statistics 2020: GLOBOCAN estimates of incidence and mortality worldwide for 36 cancers in 185 countries. CA Cancer J Clin. (2021) 71:209–49. doi: 10.3322/caac.21660, PMID: 33538338

[3] Karagiannakis DS . Systemic treatment in intermediate stage (barcelona clinic liver cancer-B) hepatocellular carcinoma. Cancers. (2023) 16:51. doi: 10.3390/cancers16010051, PMID: 38201479 PMC10778557

[4] Wu TKH Hui RWH Mak LY Fung J Seto WK Yuen MF . Hepatocellular carcinoma: Advances in systemic therapies. F1000research. (2024) 13:104. doi: 10.12688/f1000research.145493.2, PMID: 38766497 PMC11099512

[5] Qin S Bi F Gu S Bai Y Chen Z Wang Z . Donafenib versus sorafenib in first-line treatment of unresectable or metastatic hepatocellular carcinoma: a randomized, open-label, parallel-controlled phase II-III trial. J Clin Oncol: Off J Am Soc Clin Oncol. (2021) 39:3002–11. doi: 10.1200/JCO.21.00163, PMID: 34185551 PMC8445562

[6] Tutunaru CV, Alexandru DO Dracea SA Ungureanu L Popa LG Beiu C . Cabozantinib cutaneous toxicity-comprehensive review. Life (basel Switz). (2025) 15. doi: 10.3390/life15010072, PMID: 39860012 PMC11766444

[7] McLellan B Ciardiello F Lacouture ME Segaert S Van Cutsem E . Regorafenib-associated hand-foot skin reaction: practical advice on diagnosis, prevention, and management. Ann Oncol. (2015) 26:2017–26. doi: 10.1093/annonc/mdv244, PMID: 26034039 PMC4576906

[8] Nishizawa A Shinozaki E Wakatsuki T Satoh T Yamazaki N Oyamada S . Efficacy of aluminum chloride in severe regorafenib-associated hand-foot skin reactions: a single-arm trial. BMC Cancer. (2023) 23:401. doi: 10.1186/s12885-023-10864-9, PMID: 37142953 PMC10157908

[9] Chen L Wu Z Yang L Chen Y Wang W Cheng L . Nitric oxide in multikinase inhibitor-induced hand-foot skin reaction. Transl Res: J Lab Clin Med. (2022) 245:82–98. doi: 10.1016/j.trsl.2022.02.004, PMID: 35189405

[10] Said JT Singer S Iannattone L Sauder M LeBoeuf NR . Outcomes of acitretin treatment for refractory multikinase inhibitor-induced hand-foot skin reaction. JAMA Dermatol. (2022) 158:824–6. doi: 10.1001/jamadermatol.2022.1425, PMID: 35544124 PMC9096683

[11] Chanprapaph K Rutnin S Vachiramon V . Multikinase inhibitor-induced hand-foot skin reaction: a review of clinical presentation, pathogenesis, and management. Am J Clin Dermatol. (2016) 17:387–402. doi: 10.1007/s40257-016-0197-1, PMID: 27221667

[12] Ming Y Gong Y Fu X Ouyang X Peng Y Pu W . Small-molecule-based targeted therapy in liver cancer. Mol Ther: J Am Soc Gene Ther. (2024) 32:3260–87. doi: 10.1016/j.ymthe.2024.08.001, PMID: 39113358 PMC11489561

[13] Keam SJ Duggan S . Donafenib: first approval. Drugs. (2021) 81:1915–20. doi: 10.1007/s40265-021-01603-0, PMID: 34591285

[14] Cappuyns S, Corbett V Yarchoan M Finn RS Llovet JM . Critical appraisal of guideline recommendations on systemic therapies for advanced hepatocellular carcinoma: a review. JAMA Oncol. (2024) 10. doi: 10.1001/jamaoncol.2023.2677, PMID: 37535375 PMC10837331

[15] Chen R Ielasi L di Carlo A . Donafenib in hepatocellular carcinoma. Drugs Today (barc Spain: 1998). (2023) 59:83–90. doi: 10.1358/dot.2023.59.2.3507751, PMID: 36811408

[16] Jiang P Chen C Tian J Yang F Jiang ZY Hu AX . Efficacy and safety of HAIC-FOLFOX plus tyrosine kinase inhibitors and immune checkpoint inhibitors as first-line treatment for unresectable advanced hepatocellular carcinoma: a systematic review and meta-analysis. Acad Radiol. (2025) 32:4595–606. doi: 10.1016/j.acra.2024.09.061, PMID: 39384510

[17] Wu FD Zhou HF Yang W Zhu D Wu BF Shi HB . Transarterial chemoembolization combined with lenvatinib and sintilimab vs lenvatinib alone in intermediate-advanced hepatocellular carcinoma. World J Gastrointest Oncol. (2025) 17:96267. doi: 10.4251/wjgo.v17.i1.96267, PMID: 39817120 PMC11664616

[18] Zhang BH Cai YS Jiang L Yang JY . Donafenib as a first-line monotherapy for advanced hepatocellular carcinoma. Hepatobiliary Surg Nutr. (2021) 10:737–40. doi: 10.21037/hbsn-21-304, PMID: 34760990 PMC8527425

[19] Duthaler U Bachmann F Suenderhauf C Grandinetti T Pfefferkorn F Haschke M . Liver cirrhosis affects the pharmacokinetics of the six substrates of the basel phenotyping cocktail differently. Clin Pharmacokinet. (2022) 61:1039–55. doi: 10.1007/s40262-022-01119-0, PMID: 35570253 PMC9287224

[20] Hasan Alshammari A Masuo Y Fujita KI Shimada K Iida N Wakayama T . Discrimination of hand-foot skin reaction caused by tyrosine kinase inhibitors based on direct keratinocyte toxicity and vascular endothelial growth factor receptor-2 inhibition. Biochem Pharmacol. (2022) 197:114914. doi: 10.1016/j.bcp.2022.114914, PMID: 35041812

[21] Lu J Lin X Teng H Zheng Y . Atezolizumab plus bevacizumab versus lenvatinib for hepatocellular carcinoma: a systematic review and meta-analysis. J Clin Pharmacol. (2024) 64:643–51. doi: 10.1002/jcph.2402, PMID: 38311835

[22] E M V C L L L V C G M V . Hand-foot syndrome in sorafenib and lenvatinib treatment for advanced thyroid cancer. Eur Thyroid J. (2024) 13(4)e240009. Available online at: https://pubmed.ncbi.nlm.nih.gov/38954633/., PMID: 38954633 10.1530/ETJ-24-0009 PMC11301531

[23] Sun KX Cao SS Shi FH Guan Y Tang M Zhao MN . First-line treatments for advanced hepatocellular carcinoma: a network meta-analysis and cost-effectiveness analysis in China and the United States. Ther Adv Gastroenterol. (2022) 15:17562848221140662. doi: 10.1177/17562848221140662, PMID: 36518883 PMC9742927

[24] Meng R Zhang X Zhou T Luo M Qiu Y . Cost-effectiveness analysis of donafenib versus lenvatinib for first-line treatment of unresectable or metastatic hepatocellular carcinoma. Expert Rev Pharmacoeconomics Outcomes Res. (2022) 22. doi: 10.1080/14737167.2022.2079498, PMID: 35579405

[25] Zhao M Pan X Yin Y Hu H Wei J Bai Z . Cost-effectiveness analysis of five systemic treatments for unresectable hepatocellular carcinoma in China: an economic evaluation based on network meta-analysis. Front Public Health. (2022) 10:869960. doi: 10.3389/fpubh.2022.869960, PMID: 35493395 PMC9051228

[26] Guan H Wang C Zhao Z Han S . Cost-effectiveness of donafenib as first-line treatment of unresectable hepatocellular carcinoma in China. Adv Ther. (2022) 39. doi: 10.1007/s12325-022-02185-3, PMID: 35644019

[27] Fulgenzi CAM D'Alessio A Airoldi C Scotti L Demirtas CO Gennari A . Comparative efficacy of novel combination strategies for unresectable hepatocellular carcinoma: a network metanalysis of phase III trials. Eur J Cancer (oxf Engl: 1990). (2022) 174. doi: 10.1016/j.ejca.2022.06.058, PMID: 35970037

[28] Liu W Quan B Lu S Tang B Li M Chen R . First-line systemic treatment strategies for unresectable hepatocellular carcinoma: a systematic review and network meta-analysis of randomized clinical trials. Front Oncol. (2021) 11:771045. doi: 10.3389/fonc.2021.771045, PMID: 35004289 PMC8739799

[29] Han Y Zhi WH Xu F Zhang CB Huang XQ Luo JF . Selection of first-line systemic therapies for advanced hepatocellular carcinoma: a network meta-analysis of randomized controlled trials. World J Gastroenterol. (2021) 27:2415–33. doi: 10.3748/wjg.v27.i19.2415, PMID: 34040331 PMC8130040

[30] Deutsch A Leboeuf NR Lacouture ME McLellan BN . Dermatologic adverse events of systemic anticancer therapies: cytotoxic chemotherapy, targeted therapy, and immunotherapy. Am Soc Clin Oncol Educ Book. (2020) 40:485–500. doi: 10.1200/EDBK_289911, PMID: 32421446

[31] Yang CH Lin WC Chuang CK Chang YC Pang ST Lin YC . Hand-foot skin reaction in patients treated with sorafenib: a clinicopathological study of cutaneous manifestations due to multitargeted kinase inhibitor therapy. Br J Dermatol. (2008) 158:592–6. doi: 10.1111/j.1365-2133.2007.08357.x, PMID: 18070211

[32] Martin-Pozo MD Williams EA Bonnet KR Kaffenberger BH Schlundt DG Phillips EJ . Recovering from stevens-johnson syndrome and toxic epidermal necrolysis. JAMA Dermatol. (2025), e254345. doi: 10.1001/jamadermatol.2025.4345, PMID: 41222950 PMC12613091

[33] Nagpal P Bhalala M Vidholia A Sao R Sharma N Mehta D . Abdominal skin rash after TACE due to non-target embolization of hepatic falciform artery. ACG Case Rep J. (2016) 3:217–20. doi: 10.14309/crj.2016.55, PMID: 27144210 PMC4843162

[34] Calle AM Aguirre N Ardila JC Cardona Villa R . DRESS syndrome: a literature review and treatment algorithm. World Allergy Organ J. (2023) 16. doi: 10.1016/j.waojou.2022.100673, PMID: 37082745 PMC10112187

[35] Pande S . Causality or relatedness assessment in adverse drug reaction and its relevance in dermatology. Indian J Dermatol. (2018) 63:18–21. doi: 10.4103/ijd.IJD_579_17, PMID: 29527021 PMC5838749

[36] Ai L Xu Z Yang B He Q Luo P . Sorafenib-associated hand-foot skin reaction: practical advice on diagnosis, mechanism, prevention, and management. Expert Rev Clin Pharmacol. (2019) 12. doi: 10.1080/17512433.2019.1689122, PMID: 31679411

[37] Di Gion P Kanefendt F Lindauer A Scheffler M Doroshyenko O Fuhr U . Clinical pharmacokinetics of tyrosine kinase inhibitors: focus on pyrimidines, pyridines and pyrroles. Clin Pharmacokinet. (2011) 50:551–603. doi: 10.2165/11593320-000000000-00000, PMID: 21827214

[38] Mir O Coriat R Blanchet B Durand JP Boudou-Rouquette P Michels J . Sarcopenia predicts early dose-limiting toxicities and pharmacokinetics of sorafenib in patients with hepatocellular carcinoma. PLoS One. (2012) 7:e37563. doi: 10.1371/journal.pone.0037563, PMID: 22666367 PMC3364283

[39] Catalano M Casadei-Gardini A Vannini G Campani C Marra F Mini E . Lenvatinib: established and promising drug for the treatment of advanced hepatocellular carcinoma. Expert Rev Clin Pharmacol. (2021) 14:1353–65. doi: 10.1080/17512433.2021.1958674, PMID: 34289756

[40] Zschäbitz S Grüllich C . Lenvantinib: a tyrosine kinase inhibitor of VEGFR 1-3, FGFR 1-4, PDGFRα, KIT and RET. Recent Results Cancer Res Fortschr Krebsforsch Prog Dans Rech Sur Cancer. (2018) 211:187–98. doi: 10.1007/978-3-319-91442-8_13, PMID: 30069768

[41] Matsuki M Hoshi T Yamamoto Y Ikemori-Kawada M Minoshima Y Funahashi Y . Lenvatinib inhibits angiogenesis and tumor fibroblast growth factor signaling pathways in human hepatocellular carcinoma models. Cancer Med. (2018) 7:2641–53. doi: 10.1002/cam4.1517, PMID: 29733511 PMC6010799

[42] Cabanillas ME Takahashi S . Managing the adverse events associated with lenvatinib therapy in radioiodine-refractory differentiated thyroid cancer. Semin Oncol. (2019) 46:57–64. doi: 10.1053/j.seminoncol.2018.11.004, PMID: 30685073

