# Supplementary material for: Donafenib intolerance in hepatocellular carcinoma: severe hand–foot skin reaction and successful switch to lenvatinib – a case report and literature review
Source: Front Oncol. 2025 Dec 18;15:1728098. doi: 10.3389/fonc.2025.1728098 (PMC12756137; doi:10.3389/fonc.2025.1728098)
Supplement: Supplementary file 1 [file Table1.docx]

Supplementary table 1. Summary of Reported Adverse Events Associated with Donafenib

| **No.** | **Title** | **Year** | **Cancer Type** | **Adverse Reactions (concise)** | **Source** | **Relevance to HCC/TKI safety** |
| --- | --- | --- | --- | --- | --- | --- |
| 1 | Decoding Drug Interactions: Character and Degree of Pharmacokinetic Interactions Between Telmisartan and Sorafenib or Donafenib in Rats | 2025 | Preclinical (rats) | Non-clinical PK study; clinical AEs not reported | [1] | **Non-applicable (Preclinical study)** |
| 2 | Efficacy and Safety of Hepatic Artery Infusion Chemotherapy Combined with Donafenib and Camrelizumab in the Treatment of Patients with Unresectable Hepatocellular Carcinoma | 2025 | uHCC | Hypoalbuminemia 87.8%; HFSR 79.6%; ≥G3 AEs 61.2% | [2] | Applicable |
| 3 | Efficacy and safety of Donafenib plus transarterial chemoembolization and immunotherapy for hepatocellular carcinoma | 2025 | uHCC | HFSR 56.7%; rash 40%; diarrhea 16.7%; hypertension 13.3%; 3.3% discontinued ≥G3 AEs | [3] | Applicable |
| 4 | Comparison of survival benefit and safety profile between Lenvatinib and Donafenib as conversion therapy in patients with hepatocellular carcinoma | 2025 | HCC | Lenvatinib: longer OS (14.9 vs 7.9 mo) and fewer ≥G3 AEs than Donafenib | [4] | Applicable |
| 5 | A prediction model for moderate to severe pain in primary hepatic carcinoma after chemotherapy: a multi-center prospective case-control study | 2025 | PHC (post-chemo) | Not drug-specific; AEs not attributed to Donafenib | [5] | **Non-applicable (Non-drug-specific)** |
| 6 | Investigation into the Use of Surufatinib and Donafenib as Novel Multi-Kinase Inhibitors Therapeutic Agents in Managing Advanced Differentiated Thyroid Cancer: A Systematic Review | 2025 | Differentiated Thyroid Cancer | Common TKI AEs: hypertension, hand–foot syndrome (review evidence) | [6] | **Partially applicable (non-HCC)** |
| 7 | Donafenib Induces Mitochondrial Dysfunction in Liver Cancer Cells via DRP1 | 2025 | HCC (mechanistic) | Mechanistic in vitro/in vivo; no clinical AEs | [7] | **Non-applicable (Mechanistic)** |
| 8 | Donafenib combined with Sintilimab for advanced hepatocellular carcinoma: a single-arm phase II trial | 2025 | HCC | TRAEs 93.3%; ≥G3 30.0%; common: HFSR 36.7%, thrombocytopenia 30.0%, AST↑ 30.0% etc. | [8] | Applicable |
| 9 | Donafenib activates the p53 signaling pathway in hepatocellular carcinoma, induces ferroptosis, and enhances cell apoptosis | 2025 | HCC (mechanistic) | Mechanistic; no clinical AE data | [9] | **Non-applicable (Mechanistic)** |
| 10 | Absorption, metabolism, and excretion of oral [14C] radiolabeled Donafenib: an open-label, phase I, single-dose study in humans | 2024 | Healthy volunteers | Phase I ADME; generally well tolerated; non-HCC patients | [10] | **Non-applicable (Healthy volunteers)** |
| 11 | Efficacy and Safety of Targeted Therapy for Radioiodine-Refractory Differentiated Thyroid Cancer | 2025 | RR-DTC | Common AEs: hypertension, diarrhea, proteinuria, fatigue | [11] | **Partially applicable (non-HCC)** |
| 12 | VKH-like uveitis during Donafenib therapy for hepatocellular carcinoma: a case report and review of the literature | 2024 | HCC | VKH-like uveitis (rare, immune-related) | [12] | Applicable |
| 13 | Immune effect and prognosis of TACE and TKIs in patients with HCC | 2024 | HCC | Mostly mild AEs (GI symptoms, rash) | [13] | Applicable |
| 14 | Efficacy and Safety Analysis of TACE + Donafenib ± ICIs for unresectable HCC | 2024 | uHCC | Good safety; few ≥G3 AEs | [14] | Applicable |
| 15 | Adjuvant Donafenib for HCC patients at high risk of recurrence after radical resection: a real-world experience | 2024 | HCC (adjuvant) | Most AEs grade 1–2 (fatigue, mild rash) | [15] | Applicable |
| 16 | Assessment of safety and efficacy of TACE + Camrelizumab + Donafenib in HCC | 2024 | HCC | Severe AEs infrequent; mild–moderate rash/fatigue common | [16] | Applicable |
| 17 | Efficacy and safety analysis of TACE + Donafenib + Toripalimab vs TACE + Sorafenib in uHCC | 2023 | uHCC | Sorafenib group higher G3–4 hypertension (14.8% vs 4.9%); Donafenib fewer AEs | [17] | Applicable |
| 18 | Safety and efficacy of GEMOX plus Donafenib and Tislelizumab as first-line therapy for advanced biliary tract cancer | 2023 | BTC | Rash 30.8%, thrombocytopenia 15.4%, fatigue 15.4%; manageable | [18] | **Partially applicable (non-HCC)** |

AEs = Adverse events; HCC = Hepatocellular carcinoma; TKI = Tyrosine kinase inhibitor. Entries labeled “Non-applicable” or “Partially applicable” represent studies that did not include HCC patients or lacked clinical adverse-event data.

2
